# Supplementary material for: Analysis of PPARγ Signaling Activity in Psoriasis
Source: Int J Mol Sci. 2021 Aug 10;22(16):8603. doi: 10.3390/ijms22168603 (PMC8395241; doi:10.3390/ijms22168603)
Supplement: Supplementary file 1 [file ijms-22-08603-s001.zip › Supplemental materials_Analysis of PPARg signaling activity in psoriasis/Pathway models/Models images and html files/Differentiation of psoriatic T cells/38473.html]

IL23A


# Protein IL23A

|  |  |
| --- | --- |
| URN | urn:agi-llid:51561 |
| Total Entities | 0 |
| Connectivity | 767 |
| Name | IL23A |
| Description | interleukin 23 subunit alpha |
| Notes | This gene encodes a subunit of the heterodimeric cytokine interleukin 23 (IL23). IL23 is composed of this protein and the p40 subunit of interleukin 12 (IL12B). The receptor of IL23 is formed by the beta 1 subunit of IL12 (IL12RB1) and an IL23 specific subunit, IL23R. Both IL23 and IL12 can activate the transcription activator STAT4, and stimulate the production of interferon-gamma (IFNG). In contrast to IL12, which acts mainly on naive CD4(+) T cells, IL23 preferentially acts on memory CD4(+) T cells. [provided by RefSeq, Jul 2008] |
| Primary Cell Localization | Extracellular |
| Class | Ligand |

---

|  |  |
| --- | --- |
| Pathway | Th17-Cell Differentiation |
|  | Macrophage M1 Lineage |
|  | Lymphocyte Mediated Myocardial Injury in Myocarditis |
|  | Genes with Mutations Associated with Psoriasis |
|  | T-Cells Differentiation Block in Psoriasis |
|  | Dendritic Cells Function in Psoriasis |
|  | Th17-Cell Function in Systemic Lupus Erythematosus |
|  | Th17-Cell Activation in Crohn's Disease |
|  | Dendritic Cell Dysfunction in Crohn's disease |
|  | Dendritic Cell Function in Ulcerative Colitis |
|  | Nicotine anti-Inflammatory Effect in Ulcerative Colitis |
|  | Th17-Cell Differentiation in Asthma |
|  | Dendritic Cell Dysfunction in Psoriatic Arthritis |
|  | Th17-Cell and Th1 Immune Responsein Psoriatic Arthritis |
|  | Proteins Involved in Psoriatic Arthritis |
|  | Th17-Cell Numbers Reduction in HIV |
|  | CD4+ T-Cell Function Decline in HIV |
|  | Genes with Mutations Associated with Psoriatic Arthritis |
|  | Proteins with Altered Expression in Psoriatic Arthritis |
|  | IL6/IL12 Signaling Activates Immune System in Multiple Sclerosis |
|  | Immune System Role in Otitis Media |
|  | Elevated Receptors -> Expression Targets in Colon |
|  | IL23A and IL17A Provoke Cancer-Associated Inflammation |
|  | STAT3 Facilitates the Function of Treg Cells and Cancer Progression |
|  | Proteins with Altered Expression in Tumor-Promoting Inflammation |
|  | IL23R -> STAT3/NF-kB Signaling |
|  | GPCRs Family -> Expression Targets in Lymphoid System and Blood |
|  | Elevated Receptors -> Expression Targets in Bone Marrow |
|  | C5AR1 -> Expression Targets in Lymphoid System and Blood |
|  | Elevated Receptors -> Expression Targets in Breast |
|  | PPAR Psoriasis |
|  | 1\_Differentiation of psoriatic T cells |
|  | 1\_Defects in response to pathogens in the gut promotes inflammation in Crohn's disease |
|  | Differentiation of psoriatic T cells |

---

|  |  |
| --- | --- |
| Group | Genes with Mutations Associated with Psoriasis |
|  | Genes with Mutations Associated with Psoriatic Arthritis |

---

|  |  |
| --- | --- |
| MedScan ID | 51561 |

---

|  |  |
| --- | --- |
| LocusLink ID | 51561 |
|  | 83430 |
|  | 155140 |

---

|  |  |
| --- | --- |
| Alias | interleukin IL23P19 |
|  | MGC79388 |
|  | SGRF |
|  | interleukin 23 p19 subunit |
|  | interleukin 23p19 |
|  | JKA3 induced upon T-cell activation |
|  | interleukin 23, alpha subunit p |
|  | UNQ2498/PRO5798 |
|  | MGC114275 |
|  | interleukin-six, G-CSF related factor |
|  | interleukin 23 alpha subunit |
|  | interleukin 23 alpha |
|  | interleukin 23, alpha subunit p19 |
|  | IL23A |
|  | interleukin-23 subunit alpha |
|  | IL23P19 |
|  | IL-23 subunit alpha |
|  | interleukin IL23A |
|  | interleukin 23A |
|  | interleukin-23 subunit p19 |
|  | P19 |
|  | IL-23 |
|  | IL-23A |
|  | IL-23-A |
|  | IL-23p19 |

---

|  |  |
| --- | --- |
| GO ID | 0005125 |
|  | 0045519 |
|  | 0042098 |
|  | 0019221 |
|  | 0050829 |
|  | 0051607 |
|  | 0006954 |
|  | 0045087 |
|  | 0038155 |
|  | 0032693 |
|  | 1901224 |
|  | 0051135 |
|  | 0051142 |
|  | 0001916 |
|  | 0042102 |
|  | 0002827 |
|  | 2000330 |
|  | 2000318 |
|  | 0042104 |
|  | 0010536 |
|  | 0002230 |
|  | 0032725 |
|  | 0050729 |
|  | 0032729 |
|  | 0032733 |
|  | 0032735 |
|  | 0032740 |
|  | 0043382 |
|  | 0032816 |
|  | 0032819 |
|  | 0090023 |
|  | 0045672 |
|  | 0034105 |
|  | 0045944 |
|  | 0032760 |
|  | 0042531 |
|  | 0042509 |
|  | 0048771 |
|  | 0005788 |
|  | 0005576 |
|  | 0070743 |
|  | 0006955 |
|  | 0002376 |
|  | 0005615 |
|  | 0010469 |
|  | 0042346 |
|  | 0010535 |
|  | 0042517 |
|  | 0042520 |
|  | 0042523 |
|  | 0042510 |
|  | 0009615 |
|  | 0005126 |
|  | 0002295 |
|  | 0002460 |

---

|  |  |
| --- | --- |
| KEGG ID | hsa:51561 |
|  | mmu:83430 |
|  | rno:155140 |

---

|  |  |
| --- | --- |
| Organism | Homo sapiens {Organism urn:agi-taxid:9606} |
|  | Mus musculus {Organism urn:agi-taxid:10090} |
|  | Rattus norvegicus {Organism urn:agi-taxid:10116} |
|  | Mus musculus |
|  | Homo sapiens |
|  | Rattus norvegicus |

---

|  |  |
| --- | --- |
| Mouse chromosome position | 10 |

---

|  |  |
| --- | --- |
| OMIM ID | 605580 |

---

|  |  |
| --- | --- |
| Rat chromosome position | 7q11 |

---

|  |  |
| --- | --- |
| Hugo ID | 15488 |
|  | HGNC:15488 |

---

|  |  |
| --- | --- |
| Human chromosome position | 12q13.3 |

---

|  |  |
| --- | --- |
| Swiss-Prot Accession | Q9NPF7 |
|  | Q9NPF7.1 |
|  | Q9EQ14 |
|  | Q9EQ14.1 |
|  | Q91Z84 |
|  | Q91Z84.1 |
|  | Q6NZ80 |
|  | Q6NZ82 |
|  | Q9H2A5 |

---

|  |  |
| --- | --- |
| GenBank ID | NC\_000012 |
|  | XM\_011538477 |
|  | XP\_011536779 |
|  | NM\_016584 |
|  | NP\_057668 |
|  | AB030001 |
|  | BAA93687 |
|  | AC025574 |
|  | CH471054 |
|  | EAW96933 |
|  | CS498403 |
|  | CAM82731 |
|  | HI574128 |
|  | CBX47553 |
|  | JA104589 |
|  | CCA63964 |
|  | AB030000 |
|  | BAA93686 |
|  | AF301620 |
|  | AAG37232 |
|  | AJ506993 |
|  | CAD45088 |
|  | AY359083 |
|  | AAQ89442 |
|  | BC066267 |
|  | AAH66267 |
|  | BC066268 |
|  | AAH66268 |
|  | BC066269 |
|  | AAH66269 |
|  | BC067511 |
|  | AAH67511 |
|  | BC067512 |
|  | AAH67512 |
|  | BC067513 |
|  | AAH67513 |
|  | BM842783 |
|  | BM851337 |
|  | U38443 |
|  | Q9NPF7 |
|  | NC\_000076 |
|  | NM\_031252 |
|  | NP\_112542 |
|  | AC135859 |
|  | CH466578 |
|  | EDL24554 |
|  | CQ878369 |
|  | CAH59603 |
|  | FB701193 |
|  | CAS03313 |
|  | HC026803 |
|  | CBH19860 |
|  | AF301619 |
|  | AAG37231 |
|  | AK043894 |
|  | BC019953 |
|  | AAH19953 |
|  | Q9EQ14 |
|  | NC\_005106 |
|  | NM\_130410 |
|  | NP\_569094 |
|  | AC\_000075 |
|  | AAHX01047152 |
|  | AC109891 |
|  | CH474104 |
|  | EDL84876 |
|  | AY055379 |
|  | AAL18229 |
|  | BC098907 |
|  | AAH98907 |
|  | Q91Z84 |
|  | NC\_018923 |
|  | AMYH02026567 |
|  | HQ447589 |
|  | ADQ32075 |
|  | AC\_000032 |
|  | AAHY01090067 |
|  | AC\_000144 |
|  | NT\_029419 |
|  | NW\_001838059 |
|  | NW\_925395 |
|  | AC\_000055 |
|  | NT\_039500 |
|  | NW\_001030422 |
|  | NW\_001084851 |
|  | NW\_047773 |
|  | ABBA01008423 |

---

|  |  |
| --- | --- |
| Swiss-Prot ID | IL23A\_HUMAN |
|  | IL23A\_MOUSE |
|  | IL23A\_RAT |

---

|  |  |
| --- | --- |
| Cell Localization | Secreted |
|  | Extracellular |

---

|  |  |
| --- | --- |
| Ensembl ID | ENSG00000110944 |
|  | ENSP00000228534.4 |
|  | ENST00000228534.6 |
|  | ENSMUSG00000025383 |
|  | ENSMUSP00000026449.1 |
|  | ENSMUST00000026449.2 |
|  | ENSRNOG00000003254 |
|  | ENSRNOP00000004363.2 |
|  | ENSRNOT00000004363.3 |
|  | ENST00000228534.5 |
|  | ENSP00000228534 |
|  | ENST00000228534 |
|  | ENSMUSP00000026449 |
|  | ENSMUST00000026449 |
|  | ENSRNOP00000004363 |
|  | ENSRNOT00000004363 |

---

|  |  |
| --- | --- |
| MGI ID | MGI:1932410 |
|  | 1932410 |

---

|  |  |
| --- | --- |
| RGD ID | 620873 |

---

|  |  |
| --- | --- |
| Unigene ID | Hs.382212 |
|  | Mm.125482 |
|  | Hs.98309 |
|  | Rn.81073 |

---

|  |  |
| --- | --- |
| Homologene ID | 12832 |

---

|  |  |
| --- | --- |
| Shape | Rhomb |

---

|  |  |
| --- | --- |
| IPI ID | IPI00110260 |
|  | IPI00209334 |
|  | IPI00300835 |
|  | IPI00791254 |

---
